# A history of depression in patients attending a chronic pain management clinic in South Africa: A retrospective chart review

**DOI:** 10.4102/sajpsychiatry.v28i0.1673

**Published:** 2022-04-29

**Authors:** Joseph J. van Vreede, Romy Parker, Janieke van Nugteren

**Affiliations:** 1 Department of Anaesthesia and Perioperative Medicine, Faculty of Health Sciences, University of Cape Town, Cape Town, South Africa

**Keywords:** depression, chronic pain, comorbid, pain clinic, pain management

## Abstract

**Background:**

Chronic pain and depression are closely related conditions, which commonly exist as comorbid disorders. Understanding the prevalence of depression in patients presenting with chronic pain is vital for effective pain management.

**Aim:**

Our study aimed to establish the prevalence of a history of depression in patients presenting with chronic pain to a chronic pain management clinic at a tertiary academic hospital and to describe the characteristics of patients with both conditions.

**Setting:**

Groote Schuur Hospital, Chronic Pain Management Clinic, Cape Town, South Africa.

**Method:**

A retrospective review of 665 medical charts of consecutive patients accessing the clinic over a 7-year period was conducted. Baseline, patient-centred data were collected.

**Results:**

Of the 665 charts, 623 were analysed. The median age of patients was 53 years. The prevalence of depression in patients presenting with chronic pain was 32%, three times higher than the national life-time prevalence in South Africa. The majority (77%) of patients with chronic pain and depression were female (*p* < 0.01). Overall, 51% of the patients assessed were unemployed with low levels of education. The majority of our study patients had received a tricyclic antidepressant at some time prior to presentation.

**Conclusion:**

The high prevalence of a history of depression in patients presenting with chronic pain in our study, emphasises the importance of looking for and understanding the interrelation of the physiological, psychiatric, psychological and socio-economic factors that are common to both depression and chronic pain. Pain relief alone is insufficient to ensure optimal rehabilitation of these patients and integrating the management of their depression should improve patient outcomes and overall well-being.

## Introduction

Chronic pain is an extremely complex and poorly understood medical condition that may result in a patient repeatedly seeking medical care. This condition can have profound effects on a patient’s physical and mental well-being and on their ability to function daily.^1^ Chronic pain may be more common in individuals with depressive disorders and the two conditions appear to be closely related.^2^ Depression can cause pain – and pain can cause depression. Both are debilitating conditions that exist as comorbid disorders in as many as 80% of patients attending specialist pain units.^3^ Pain and depression share biological pathways and neurotransmitters with recent studies suggesting overlaps between pain- and depression-induced neuroplastic and neurobiological changes.^4^ Therefore, the successful management of a patient’s chronic pain, requires the clinician to assess for and treat any underlying clinical depression.^5^

The prevalence of depression in patients with chronic pain is described from 4.7% to 22% in population-based studies and estimated to be between 6% and 46% in studies from the primary care sector.^2^ In a study published in 2016 evaluating people with chronic pain attending a specialist pain clinic in the United Kingdom, 60.8% (95% confidence interval [CI]: 58.0–63.6) met the criteria for probable depression whilst 33.8% met the threshold for severe depression on the 9-item Patient Health Questionnaire. Multivariate analysis showed a positive association between severe depression and an increase in total healthcare costs as compared with patients without depression.^2^ In a recent systematic review and meta-analysis, Kleykamp et al. found that a quarter of all patients with fibromyalgia had major depressive disorder (MDD) and more than 50% of all fibromyalgia patients experienced a MDD during their lifetime.^6^ This was confirmed by Løge-Hagen et al., who in their systematic review describing the prevalence of psychiatric and chronic pain comorbidities in fibromyalgia, found the weighted prevalence of MDD in patients with fibromyalgia to range from 52% to 63%.^7^

In the first nationally representative psychiatric epidemiological survey, the South Africa Stress and Health (SASH) study indicated that there is an elevated risk of mental health disorders in South Africa (SA) compared with several other high- and middle-income countries.^8^ Major depressive disorder, agoraphobia and alcohol abuse were the most prevalent 12-month Diagnostic and Statistical Manual of Mental Disorders-IV (DSM-IV) and Composite International Diagnostic Interview (CIDI) disorders in the sample population. The prevalence of major depression was 9.7% for lifetime and 4.9% for the 12 months prior to the interview. In a recently published study conducted in a large nationally representative sample, the prevalence of chronic pain in South Africa was described at 18.3% (95% CI: 17.0–19.7).^9^ There are, however, a paucity of studies describing the co-prevalence of chronic pain and depression in the sub-Saharan African context. Cross-national data from 17 countries in Europe, the Americas, the Middle East, Africa, Asia and the South Pacific based on large population surveys of community-dwelling adults, revealed that mental disorders are more common amongst persons with back and neck pain than amongst persons without. Pooled odds ratios were 2.3 (95% CI: 2.1–2.5) for mood disorders and 2.2 (95% CI: 2.1–2.4) for anxiety disorders in people with, versus without chronic back and neck pain. Mental health disorders were associated with chronic back and neck pain and this pattern was consistently evident across both developed and developing countries.^10^

Literature suggests that socio-demographic risk factors for the development of chronic pain include being female, of older age, lower socio-economic status, geographical and cultural background, employment status, occupational factors and having a history of abuse or interpersonal violence. These factors may overlap with the risk of development of depression.^11,12^ South Africa is a country with significant socio-economic inequalities; which translates to health system inequalities and negatively impacts health.^13^ Therefore, in a country where the majority of people live in low-resource settings with high stressors and poor access to adequate healthcare services; they may be at an increased risk of both depression and chronic pain.

The setting for our study was the Groote Schuur Hospital (GSH) Chronic Pain Management Clinic (CPMC), a specialist, interdisciplinary clinic that provides outpatient consultation and treatment for patients with chronic pain, described as pain experienced on most days for a period of three months or longer.^14^ There is a two-tier healthcare system in South Africa with a large subsidised public sector and a small private sector, funded through individual contributions to medical aid schemes or health insurance. The public sector is state-funded and caters to the majority – approximately 71% – of the population and has a five-layered structure, which was developed to provide cost effective healthcare to all citizens, namely primary healthcare (clinic-based); secondary level district and regional hospitals; tertiary (academic) hospitals; central (academic) hospitals and Specialised hospitals. Tertiary, central and specialised hospitals rely on a referral process. Generally, the referral population originates from specialist units at GSH including general surgery, orthopaedics, rheumatology and neurology services and patients who have sought previous care at primary and secondary healthcare facilities. Most referrals are from Cape Town, Western Cape, South Africa.

Understanding the prevalence of depression in the Pain Clinic population in South Africa and its relationship to patient characteristics is beneficial to clinicians managing these patients, to the patients themselves and to stakeholders. Acknowledgement of the risk of depression in patients presenting with chronic pain is imperative to effectively managing these patients. It is important for clinicians to understand that pain relief alone may be insufficient to ensure optimal rehabilitation and that the management of underlying depression should be integrated into the treatment plan. This information is essential for stakeholders as staff and resource allocation to pain management services need to be sufficient to provide psychiatric and psychological input. Chronic pain in association with depression should therefore be considered a serious condition and a risk marker requiring intensive management to minimise the detrimental impact on life and health.^15^

## Aim and objectives

The aims of our study were to (1) establish the prevalence of a history of depression in patients presenting with chronic pain to the CPMC at GSH over a 7-year period and (2) describe the co-prevalence of chronic pain and depression by patient characteristics, namely age, sex, income status and the use of recreational drugs, tobacco or alcohol.

## Research methods and design

### Study design

A retrospective review of 665 medical charts of consecutive patients who accessed the CPMC at GSH, over a specified period, was conducted.

### Sample

The sampling frame included all patients attending an initial assessment between June 2010 and June 2017. In June 2010, a clerking form that required documentation of a patient’s history of depression to be captured on admission to the CPMC was implemented. Medical folders were excluded if the admission data captured by the clerking doctor were missing or incomplete.

### Data collection

We collected baseline, patient-centred data that was manually recorded in the admission notes as part of the routine self-reported history obtained on the initial assessment of the patient at the clinic. A standardised data collection form was used for data extraction and the following data were collected: age; sex; employment and income status; use of recreational drugs, tobacco and alcohol; details of the referring team, patient-reported and clinician-reported (from the referral letter) history of depression (or other psychiatric conditions), the current or previous use of antidepressant medication and the admission pain score. The latter included an assessment of pain intensity or severity, which is modelled on the Brief Pain Inventory (BPI) and determined pain on admission using a numeric scale ranging from 0 (‘no pain’) to 10 (‘pain as bad as you can imagine’).^16^ The documented pain interference scale characterised the degree of pain-related functional impairment using scales from 0 (‘does not interfere’) to 10 (‘completely interferes’).

To protect patient anonymity all data extraction was performed by the principal investigator who is a registered healthcare professional, trained in good clinical practice. The ethical principles of patient confidentiality were protected throughout by anonymising data at the source. Data were collated into Microsoft Excel 2013 for analysis. Data were subject to quality control procedures by an external auditor who performed a 10% quality control check of the folders assessed, the data collection forms and the data entry procedure.

### Data analysis

Statistical analysis was performed using statistical software Statistica (Version 13.1, April 2016, Germany). A non-parametric approach to analysis was adopted based on the categorical nature of most of the data. In addition, patient ages were not normally distributed and were analysed using a non-parametric approach. Numerical data are presented as medians (interquartile range), categorical data as frequencies (with percentages). Differences in numerical data were compared using the Mann–Whitney U test. Differences in distribution of categorical data were tested using Spearman’s rank order correlations. Significance was accepted throughout as *p* < 0.05.

### Ethical considerations

Our study was approved by the Human Research Ethics Committee of the Faculty of Health Sciences at the University of Cape Town (HREC REF: 505/2018) and the Departmental Research Committee of the Department of Anaesthesia and Perioperative Medicine at Groote Schuur Hospital. The principles of the Declaration of Helsinki were adhered to throughout.

## Results

A total of 665 consecutive patient folders were reviewed for the period June 2010–June 2017, of which 42 were excluded because of incomplete data. Thus, 623 patient folders were included in the analysis.

### Baseline socio-demographic patient characteristics

The median (interquartile range [IQR]) age of the patients was 53 (44–63) years with 428 (69%) being female. At the time of initial assessment at the CPMC, the majority of patients were unemployed (315; 51%) and of those whose level of education was determined (*n* = 529), 328 (62%) had not completed 12 years of schooling. In addition, 139 (22%) were receiving either state funded or privately funded disability grants (Table 1).

**TABLE 1:** Sociodemographic profile of the patients (*n* = 623).

Characteristics	Frequency	
	*n*	%
**Sex**		
Female	428	69
Male	195	31
**Level of education**		
No formal education	7	1
Grade 1–7	111	18
Grade 8–11	210	34
Grade 12	152	24
Post-schooling	49	8
Unknown	94	15
**Employment status**		
Employed	158	25
Unemployed	315	51
Pension	150	24
**Disability grants (DG)**		
Receiving DG	139	22
Not receiving DG	484	78

### Patient health profiles

Overall, surgical disciplines accounted for 402 (65%) of the referrals, with referrals from the Department of Orthopaedics (248; 40%) predominant (Table 2). Recreational substance use in the form of tobacco, alcohol or cannabis was reported by 266 (43%) of the patients. The majority of whom were using tobacco (227; 85%) with 226 (85%) using one substance only, 34 (13%) using two substances and five (2%) patients reporting use of three substances.

**TABLE 2:** Sources of referral and recreational drug use.

Sources	*n*	%
**Hospital disciplines referring patients to the CPMC (*n* = 623)**		
Surgical	402	64.5
Medical	184	29.5
Allied health	19	3
Psychiatry	18	3
**Recreation drug use (*n* = 266)**		
Tobacco alone	188	71
Alcohol alone	35	13
Cannabis alone	3	1
Tobacco and Alcohol	31	12
Tobacco and/or Alcohol and Cannabis	9	3

CPMC, chronic pain management clinic.

A BPI was completed by all patients on admission to the CPMC. The median (IQR) scores on the BPI for pain severity scores (PSS) were 8 (6.5–9.0) and 8 (6.0–9.0) for the pain interference with function score (PIS).

### Mental health profile of the patients

**FIGURE 1:**
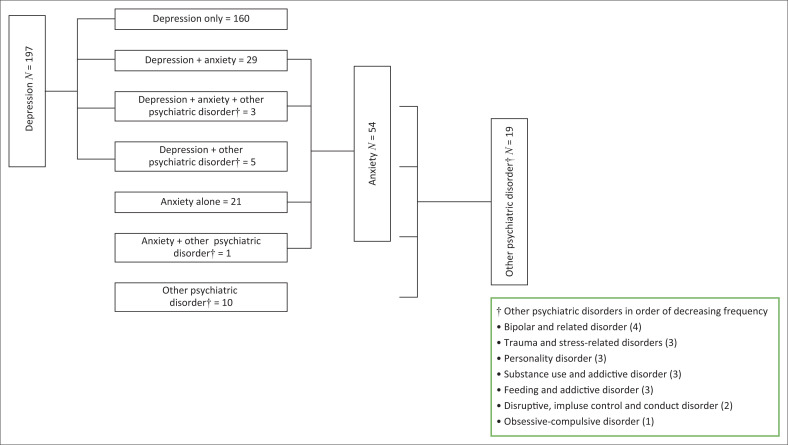
Mental health profiles of patients reporting a history of being diagnosed with depression (*n* = 197), anxiety and other psychiatric disorders.

In the 623 folders reviewed, 197 (32%) of the patients provided a history of being diagnosed with depression. Of these, 160 were diagnosed only with depression, 29 had comorbid anxiety with depression and a further three had depression, anxiety and one other psychiatric condition. In total, 229 (37%) of all patients reported a history of being diagnosed with one or more psychiatric disorder, with depression accounting for 86% of the 229 (Figure 1). The diagnosis of having a history of a mental health condition was most commonly made by a healthcare professional (164; 71%) as evidenced by a referral letter, with 66 (29%) of the patients self-reporting a previous diagnosis made by a healthcare professional. This information could not be verified except by noting the medication that the patient had received in the past.

Patients were receiving a range of central nervous system (CNS) acting agents (Table 3), which may have been prescribed for these mental health conditions or as part of the multimodal pharmacotherapy in the management of persistent or chronic pain. The most common of these included the tricyclic antidepressant agent, amitriptyline; selective serotonin reuptake inhibitors (SSRIs) and the gabapentanoid, pregabalin, which has been licensed for the treatment of painful diabetic peripheral neuropathy and postherpetic neuralgia.^17^ Several patients were receiving therapeutic doses of more than one medication from the same class or from different classes (multiclass polypharmacy) of psychotropic drugs. Polypharmacy is a common clinical practice for many psychiatric and pain conditions.^18^

**TABLE 3:** Central nervous system agents previously or currently used in patients seen at the chronic pain management clinic.

Central nervous system agents previously or currently used	*n*	%
**Antidepressants**		
Tricyclic antidepressants (TCAs)	320	51
Selective serotonin reuptake inhibitors (SSRIs)	71	11
Serotonin-norepinephrine reuptake inhibitors (SNRIs)	30	5
Tetracyclic antidepressants (TeCAs)	6	1
Other (s-erotonin antagonist and reuptake inhibitors, norepinephrine-dopamine reuptake inhibitor)	4	0.6
**Anticonvulsants**		
Pregabalin	84	13
Carbamazepine	42	7
Gabapentin	9	1
Sodium valproate	4	0.6
Lamotrigine	3	0.5
**Anxiolytics, sedatives and hypnotics**		
Benzodiazepines	39	6
Imidazopyridine	8	1
Zopiclone	8	1
Anti-psychotic agents	4	0.6
Other: CNS stimulants, cholinergic agonists, anti-parkinsonian agents	9	1

CNS, central nervous system.

### Baseline characteristics across the two clinically defined groups

A comparison between two groups: patients with chronic pain and a history of depression (PD) and patients with chronic pain only (P), with no history of depression was performed to explore the interactions between certain baseline characteristics.

As shown in Table 4, Spearman’s analysis revealed that the female-to-male ratio of patients in the PD group was higher (*p* < 0.01), with more women giving a comorbid history of depression with chronic pain than men. There was no difference between the two groups with respect to age, employment status and the level of education received.

**TABLE 4:** Comparison of baseline characteristics between patients with chronic pain only (P) and patients with chronic pain and a history of depression (PD).

Characteristics	Chronic pain with no history of depression (P) (*n* = 426)				Chronic pain with history of depression (PD) (*n* = 197)				Statistical test
	*n*	%	IQR	ratio	*n*	%	IQR	ratio	
**Sex**									Spearman’s *R* = −0.13; *p* < 0.01*
Female	276	65	-	-	152	77	-	-	-
Male	150	35	-	-	45	23	-	-	-
Female to male	-	-	-	1.8 : 1	-	-	-	3.4 : 1	-
**Age**	54	-	45–65	-	52	-	42–61	-	*U* = 38 299; *p* = 0.08
**Employment**									Spearman’s *R* = −0.03; *p* = 0.44
Employed	112	26	-	-	46	23	-	-	-
Unemployed	201	47	-	-	114	58	-	-	-
Pension	113	27	-	-	37	19	-	-	-
**Disability grant**									Spearman’s *R* = 0.07; *p* = 0.09
Receiving DG	87	20	-	-	52	26	-	-	-
Not receiving DG	339	80	-	-	145	74	-	-	-
**Level of education**									Spearman’s *R* = 0.06; *p* = 0.13
No formal education	5	1	-	-	2	1	-	-	-
Grades 1–7	82	19	-	-	29	15	-	-	-
Grade 8–11	149	35	-	-	61	31	-	-	-
Grade 12	96	23	-	-	56	28	-	-	-
Post-schooling	25	6	-	-	24	12	-	-	-
Unknown	69	16	-	-	25	13	-	-	-
**Recreational drug use**									
Tobacco alone	130	31	-	-	58	29	-	-	-
Alcohol alone	22	5	-	-	13	7	-	-	-
Cannabis alone	3	0.7	-	-	0	-	-	-	-
Tobacco and alcohol	19	4	-	-	12	6	-	-	-
Tobacco and/or Alcohol and Cannabis	5	1	-	-	4	2	-	-	-
**Average pain scores**									
Pain severity score (PSS)	7.6				7.7				*U* = 40 591; *p* = 0.51
Pain interference core (PIS)	7.3				7.7				*U* = 37 792; *p* = 0.05

DG, disability grants; IQR, interquartile range.

* , Indicates significant difference between groups at *p* < 0.05.

## Discussion

We found a high prevalence of comorbid pre-existing depression in patients presenting with chronic pain to our specialised CPMC (32%). This is more than three times higher than the described life-time prevalence in South Africa of 9.7%.^8,19,20^ The prevalence of depression in patients with chronic pain varies widely in population-based studies, however, our results are in keeping with a 2016 publication evaluating people with chronic pain attending a specialist pain clinic in the United Kingdom, where 33.8% of attendees met the threshold for severe depression.^2^

A World Health Organization (WHO) report predicted that MDD will become the leading cause of disability in the world by 2030.^21^ The organisation also emphasises the significance of the relationship between mental and physical health.^22^ In their World Health Survey, they reported that the:

[*C*]omorbidity of depression with a chronic illness incrementally worsens health outcomes in comparison to depression alone, any chronic disease alone, or any combination of chronic diseases in the absence of depression.^20^

Accumulating evidence suggests than neuroinflammation plays a critical role in the pathogenesis of both depression and chronic pain.^23^ Apart from the common pathophysiological mechanisms, however, these two entities have been thought to have several other social, demographic and clinical links, which we aimed to explore.

Of significance, we found that 77% of patients with chronic pain plus a history of depression were female compared with 23% of male patients with chronic pain and a history of depression (*p* < 0.01). In the Global Burden of Disease Study 2017, the annual prevalence of major depression was found to be higher in women at 4.1% compared with 2.7% in men, representing a 1.5-fold greater incidence in women.^24,25^ Women have a higher risk of depression than men, particularly during puberty, prior to menstruation, during pregnancy and the postpartum period and at perimenopause. In South Africa it is quoted that one in three women during or after childbirth, experience a mental health disorder, primarily depression.^26^ This figure is threefold higher than the WHO described worldwide prevalence.^27^ The mechanisms that appear to contribute to women’s vulnerability to depression may overlap with those for chronic pain. In the peripartum periods, besides the physiological hormonal changes present, multiple life changes contribute to vulnerability.^28^ The low levels of oestrogen during the transition to menopause, either biologically or abruptly after a hysterectomy, combined with acute sleep disruptions and inherited traits account for the increased prevalence of depression in this group of patients. In South Africa, women may also be vulnerable to domestic abuse, both physical and emotional and may be victims of sexual or racial discrimination on a social level, affecting their self-esteem and contributing to their vulnerability to depression. A further social factor contributing to women’s susceptibility is poverty, with studies showing that women are more likely than men to live in poverty. All these female hormonal fluctuations that have been implicated in depression^29^ have also been implicated in the development of chronic pain. However, other biological and genetic factors, plus personal life events or triggers are also associated with a risk of both depression and chronic pain. Women in South Africa play an important role in the nation’s economy and an essential domestic role at home with their families, so this finding merits more attention to ensure women’s overall physical health and mental well-being.

The majority of our study patients had received amitriptyline, followed by SSRIs. Amitriptyline is the most commonly used antidepressant in South Africa, likely because of its low cost and accessibility in primary healthcare facilities. A total of 51% of our study patients had previously received or were currently on amitriptyline – either to help with sleep, chronic pain management – specifically neuropathic pain and at higher doses for the treatment of depression. Tricyclic antidepressants are used in the management of chronic pain and for sleep and are used in the management of various forms of pain, including cancer pain,^30^ fibromyalgia^31^ and neuropathic pain.^32^ The exact mechanism of its action for pain remains uncertain, although it is known to inhibit both serotonin and noradrenaline reuptake.

Serotonin-norepinephrine reuptake inhibitors are more commonly advised for the treatment of chronic pain because of the effect of norepinephrine on pain control, but studies have emphasised that it is the balance between serotonin, norepinephrine, other neurotransmitters and receptor functions that produce the inhibition of descending pain pathways and central pain processing.^33,34^ However, the second most commonly used antidepressant in our study was the SSRI fluoxetine. Although the evidence suggests that SSRIs, are effective in low back pain and whiplash-associated disorder,^35^ these drugs do not appear to have a specific analgesic effect. It appears that SSRIs reduce the impact of chronic pain by reducing depression rather than via a reduction of pain severity. The large proportion of patients in our study who were receiving SSRIs may have been prescribed the medication to target their mood disorder as a primary condition with the comorbid pain disorder being managed as a secondary or separate condition. Other reasons for the high prescribing rate of SSRIs may be clinician familiarity with and ease of access to fluoxetine and possibly less familiarity with the mechanism of action of SSRIs when treating multiple or comorbid conditions. Currently, SNRIs in our setting require authorisation and special release by the psychiatry department.

There has been a clear paradigm shift when treating chronic pain syndromes, from using solely opioid-based treatment plans to multimodal management models. In addition, treating comorbid conditions particularly in the elderly, may impact the clinical outcomes in patients presenting with chronic pain. The median age of patients presenting to the pain clinic was 53 years and we were unable to show a significant age difference between those with and without depression. But a proper assessment for depression in older adults is important as it is estimated that 13% of the elderly population will suffer simultaneously from the two conditions.^23^ Current literature suggests that when pharmacotherapy is considered in the management of this cohort, that combined therapy focusing on both their depression and chronic pain, may yield superior outcomes than managing either condition in isolation.^36,37^

Most of the patients accessing our pain clinic come from low socioeconomic groups, and our results showed that overall, 51% of the patients assessed were unemployed with low levels of education. There was no association between those with and without depression with respect to employment status and the level of education obtained, but this was not unexpected as the majority of patients accessing our public pain clinic are unemployed with low levels of education. People with lower levels of income and education are at an increased risk of depression^38^ and chronic pain.^39^ In general, low socioeconomic status is associated with a high psychiatric morbidity, more disability and poorer access to healthcare. The lack of access to healthcare services in these communities, means both depression and pain may go undiagnosed, management and follow-up is likely to be suboptimal and appropriate referrals are delayed.

The relationship between education and pain and depression throughout adult life is unclear. Education may influence socioeconomic status such as occupation and income. However, the Trøndelag Health Study (HUNT study) supports the notion that ‘higher educational level or the factors that are reflected by higher education, may protect against anxiety and depression’, with the protective effect accumulating throughout life.^40^ South Africa is considered a low- and middle-income country where many patients have low levels of education and live in poverty. These factors increase their risk of developing both chronic pain and depression with the added challenge of limited mental healthcare resources negatively affecting potential recovery.

Our study has numerous limitations associated with retrospective chart reviews and our study relied on accurate information being captured on admission to the CPMC via a qualitative interview process. The patients reflected on their condition but are inherently subject to recall and response biases. The major limitation of our study was reliance on the patient self-reporting a history of depression and this information was not being routinely classified by a specialist using the DSM-V. This method lacks the depth and diagnostic accuracy of a clinical assessment by a trained healthcare professional and the validity of not using the gold standard clinical interview as opposed to a retrospective chart review means that our study may both miss possible undiagnosed cases and erroneously identify or classify cases as patients with depression. In addition, the calculated prevalence in this group of chronic pain patients may be subject to inclusion bias because of preferential referral of patients with greater pain and disability who get referred to a specialist clinic at a tertiary academic hospital. Given these inconsistencies, we recommend further studies in this field be performed in a prospective manner, with the utilisation of a psychiatrist or a clinical psychologist, to accurately classify patients according to their mental health status. Research is also required to determine which treatment approaches would be most successful in managing chronic pain and depression in these patients; whether psychological treatment with or without pharmacological interventions is effective and which combinations and dosages would be the best to improve the physical and mental health outcome of the patient in a cost-effective manner that would be sustainable in the South African setting.

## Conclusion

The prevalence of a history of depression in patients presenting with chronic pain to our pain clinic was found to be 32%, which is three times higher than the national lifetime prevalence in South Africa. In the last decade, the significant role of depression in chronic pain syndromes has been emphasised; acknowledging that chronic pain and depression share neurobiology and neuroanatomy. Our study provides essential insight into the importance of psychiatric and psychological management as an integral part of an effective pain management strategy and the need for an interdisciplinary team approach in chronic pain management. Our findings emphasise the importance of the interrelation of the physiological, psychiatric, psychological and socio-economic factors that link depression and chronic pain. Pain relief alone is insufficient to ensure optimal rehabilitation and integrating the management of depression using pharmacological and non-pharmacological treatment modalities with an interdisciplinary approach should be utilised to improve patient outcomes and overall well-being.
